# Cytotoxic, Antitumor and Toxicological Profile of *Passiflora alata* Leaf Extract

**DOI:** 10.3390/molecules25204814

**Published:** 2020-10-20

**Authors:** Ricardo G. Amaral, Silvana V. F. Gomes, Luciana N. Andrade, Sara A. dos Santos, Patrícia Severino, Ricardo L. C. de Albuquerque Júnior, Eliana B. Souto, Geraldo C. Brandão, Sandra L. Santos, Jorge M. David, Adriana A. Carvalho

**Affiliations:** 1 Department of Physiology, Federal University of Sergipe, São Cristóvão, Sergipe 49100-000, Brazil; ricardoamaral23@hotmail.com (R.G.A.); sara_querque@yahoo.com.br (S.A.d.S.); sandralauton@gmail.com (S.L.S.); 2 Institute of Technology and Research, University of Tiradentes, Aracaju, Sergipe 49032-490, Brazil; svfloresta@hotmail.com (S.V.F.G.); pattypharma@gmail.com (P.S.); ricardo.patologia@uol.com.br (R.L.C.d.A.J.); 3 Department of Medicine, Federal University of Sergipe (UFS), Avenida Governador Marcelo Déda, Lagarto-SE 49400-000, Brazil; 4 Center for Biomedical Engineering, Department of Medicine, Brigham and Women′s Hospital, Harvard Medical School, 65 Landsdowne Street, Cambridge, MA 02139, USA; 5 Tiradentes Institute, 150 Mt Vernon St, Dorchester, MA 02125, USA; 6 Department of Pharmaceutical Technology, Faculty of Pharmacy, University of Coimbra, Pólo das Ciências da Saúde, Azinhaga de Santa Comba, 3000-548 Coimbra, Portugal; 7 CEB—Centre of Biological Engineering, University of Minho, Campus de Gualtar, 4710-057 Braga, Portugal; 8 Department of Pharmacy, Federal University of Ouro Preto, Ouro Preto 78950-000, Brazil; celiobrandao@ufop.edu.br; 9 Institute of Chemistry, Federal University of Bahia, Salvador 40000-000, Brazil; jmdavid@ufba.br

**Keywords:** natural products, cytotoxicity, antitumor activity, cancer, *Passiflora alata*

## Abstract

*Passiflora alata* or passion fruit is a native flowering plant from Amazon, geographically spread from Peru to Brazil. The plant has long been used in folks medicine for its pharmacological properties and is included in the Brazilian Pharmacopoeia since 1929. The aim of this study was to evaluate the potential cytotoxic and antitumor activities of *Passiflora alata* leaf extract (P*a*LE) in S180-tumor bearing mice. The percentage of cell proliferation inhibition (% CPI) and IC_50_ in relation to 4 tumor cell lines were determined in PC3, K-562, HepG2 and S180 cell lines using the MTT assay. P*a*LE showed a CPI > 75% and greater potency (IC_50_ < 30 µg/mL) against PC3 and S180 cell lines. *Pa*LE showed antitumor activity in treatments intraperitoneally (36.75% and 44.99% at doses of 100 and 150 mg/kg/day, respectively). Toxicological changes were shown in the reduced body mass associated with reduced food consumption, increased spleen mass associated with histopathological increase in the white pulp of the spleen and increased number of total leukocytes with changes in the percentage relationship between lymphocytes and neutrophils. Our outcomes corroborate the conclusion that *Pa*LE has antitumor activity in vitro and in vivo with low toxicity.

## 1. Introduction

More than 100 types of cancers have already been characterized, for which some type of systemic or local treatment has been proposed for curative or palliative purposes [1,2]. However, systemic chemotherapy is still the most widely used, together with local therapies, such as surgery and/or radiation therapy. Although chemotherapeutic drugs in general are effective in destroying neoplastic cells, the major challenge is the distinction between neoplastic and normal cells. This lack of specificity of chemotherapeutic drugs is a serious limiting factor, due to several adverse effects such as diarrhea, vomiting, nausea, alopecia and greater risk of serious infections [3,4].

This risk of infections is mainly caused by hematological changes and more specifically leukopenia and percentage changes in the lymphocyte and neutrophil ratio in the differential count. This information was confirmed by Britto et al. [5], in which a drastic decrease in the levels of total leukocytes and a change in the percentage ratio of lymphocytes and neutrophils were observed in the group treated with antineoplastic. As a consequence of hematological changes, alteration in the mass of lymphoid and histological organs can be observed.

Even with these existing treatment modalities, cancer causes more deaths than all coronary heart disease or strokes [6,7,8]. In this regard, investigating more effective and selective compounds that reduce this growing problem is a challenge, and nature is an alternative to this problem [9].

Newman and Cragg [10] revised the list of therapeutic agents approved worldwide between 1981 and 2014 for all diseases, and from 1950 to 2014 for cancer treatment. A total of 136 cancer drugs were registered worldwide, with only 17% being of synthetic origin whereas 83% were natural products or from natural-based sources. A discovery that amplifies the relevance of research with natural products is the one of the most famous antineoplastic drugs, paclitaxel, isolated from the bark of the *Taxus brevifolia* tree and used in the treatment of breast cancer [9].

Despite the success of these natural products, most plant species worldwide have not been systematically investigated in drug discovery campaigns [11]. Within this universe, biological activities of these compounds especially with respect to cytotoxicity in tumor cell lines are still limited [12], the aim of our work has been the search for new antineoplastic drugs derived from natural products, with a special interest on the wide diversity of the genus *Passiflora* (P.). Some species, such as *P. edullis* Sims [13], *P. ligularis* Juss [14], *P. incarnata* [15,16,17], *P. molissima* [18] and P. *tetandra* [19] have shown cytotoxic activity against tumor cell lines, and exhibit chemopreventive or antitumor activity in vivo.

Amaral et al. [20] carried out for the first time a study on the cytotoxic potential of the leaf extract of 14 species of the genus *Passiflora*, against cancer cell lines. Of these, only the *Passiflora alata* leaves extract (*PaLE*) showed relevant cytotoxic potential. Based on these data, the hypothesis that besides the tested 14 species, other *Passiflora* species could show anti-cancer activity is emphasized [20].

*P. alata* has commercial importance for its food consumption, as well as a herbal medicine due to several pharmacological properties [21]. Besides, the genotoxic potential of *P. alata* reported by Boeira et al. [22] raised the question whether the species may also present antitumor activity in experimental biological models. In this work, we have characterized the in vitro cytotoxic profile of *PaLE* against four tumor cell lines and its in vivo antitumoral and toxicological effects in sarcoma tumor 180-bearing mice.

## 2. Results and Discussion

The main groups of constituents of *Passiflora alata* leaves are flavonoids and saponins [9]. As the pharmacological activities of natural products are directly linked to their chemical composition, and the *Pa*LE obtained by the accelerated solvent extraction method (ASE) has identified the presence of flavonoids [20], we decided to investigate the presence of saponins in the extract through ultra-efficient liquid chromatography. Table 1 shows the identified compounds.

Most of the secondary metabolites identified in *Pa*LE (flavonoids and saponins) have proven cytotoxic or anticancer activities, emphasizing the hypothesis that *Pa*LE in vitro action is possibly mediated by synergistic activity among its constituents. Based on these data, the present study advances tests on the anti-tumor potential of *Pa*LE.

The next step was the profiling of cytotoxic activity of the extract against four tumor cell lines (PC-3, K-562, HepG2, and S180) using the MTT method. The data obtained in this study (Table 2), indicate that *Pa*LE inhibits cell proliferation (CPI) > 75% against the four tested tumor cell lines and IC_50_ < 30 µg/mL for two tumor cell lines (PC-3 and S180). Although *Pa*LE showed high cytotoxic activity in vitro, it may be considered in the development of anticancer drugs against PC-3 and S180, for which the IC_50_ value was reported to be below 30 µg/mL.

**Figure 1:**
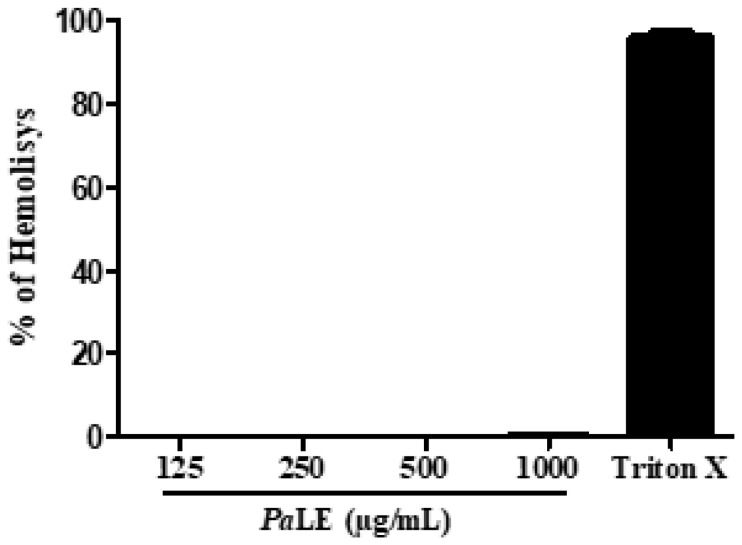
Evaluation of *Pa*LE hemolytic activity. The data are presented as mean ± standard deviation of three independent experiments.

*Pa*LE was thus subjected to an in vitro assessment of hemolytic activity against erythrocytes. In this assay, insignificant hemolytic activity (0.40 ± 0.25%) was observed at concentrations of 1000 µg/mL. At concentrations of 125, 250, and 500 µg/mL, no hemolytic activity was observed (Figure 1). We therefore anticipate that *Pa*LE has insignificant hemolytic activity due to the presence of saponins, enhanced by the protection offered by the presence of flavonoids in the composition of the extract.

**Figure 2:**
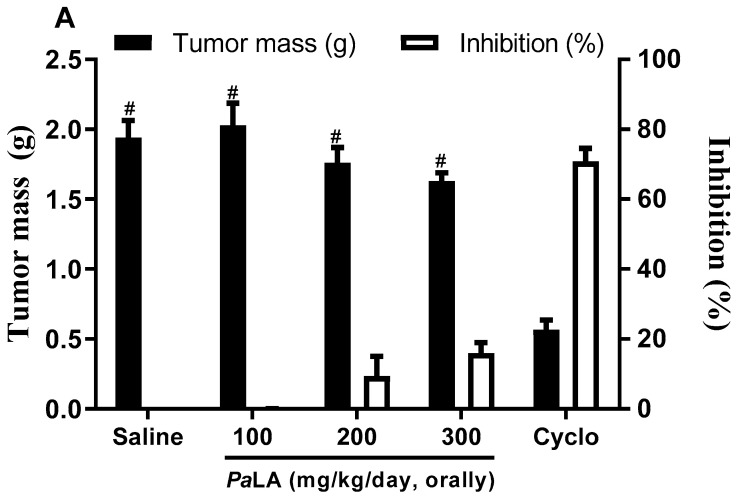

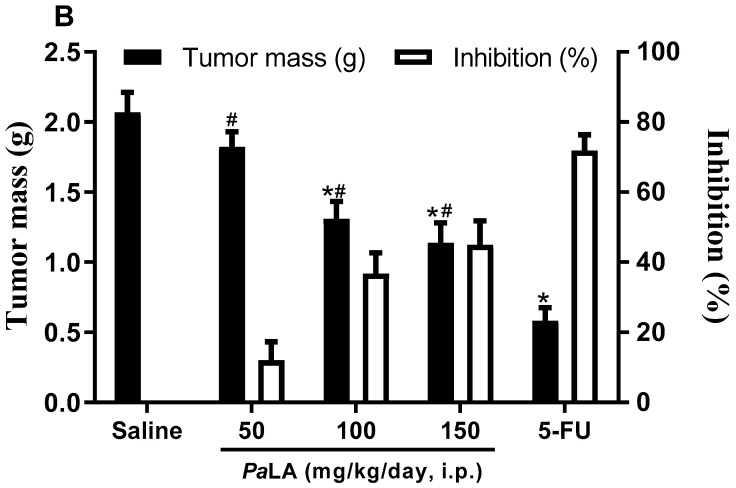
The effect *of Pa*LE in mice inoculated with S180 cells. The graphs show the tumor mass in grams (g) and degree of growth inhibition (%). The animals were treated orally (**A**) and i.p. (**B**), starting one day after the tumor implantation, for 7 consecutive days. Saline was used as a negative control. 5-FU 25 mg/kg/day and Cyclophosphamide 15 mg/kg/day (cycle) were used as a positive control. Data are presented as mean ± standard error of the mean (*n* = 7–10 animals/group) and the results evaluated by one-way analysis of variance (ANOVA) with a Student Newman Keuls post-test. * *p* < 0.05 compared to the saline group, # *p* < 0.05 compared to the 5-FU or Cyclo group.

Since *Pa*LE is cytotoxic for tumor cell lines, with insignificant activity against non-tumor cells, we have progressed to the evaluation of the antitumor activity in vivo, upon the i.p. and oral administration in an experimental S180 tumor mice model. No significant in vivo antitumor activity was observed upon oral administration of the *Pa*LE (Figure 2A). However, after i.p. administration, the extract inhibited tumor growth by 12.05, 36.75 and 44.99% at doses of 50, 100 and 150 mg/kg/day, respectively. In the *Pa*LE-treated groups, the mean tumor mass was 1.83 ± 0.11 g, 1.31 ± 0.12 g, and 1.14 ± 0.14 g for doses of 50, 100, and 150 mg/kg/day, respectively. There was only a significant reduction (*p* < 0.05) in the mass of tumors of animals treated with 5-fluorouracil and *Pa*LE at doses of 100 and 150 mg/kg/day when compared to the saline group. The three groups treated with *Pa*LE showed significant changes when compared to the positive control group (Figure 2B). Thus, the results show that *Pa*LE at doses of 100 and 150 mg/kg/day has antitumor activity in a S180 mice model. We observed the in vivo antitumor activity of *Pa*LE after i.p. at doses of 100 and 150 mg/kg/day (Figure 2B).

**Figure 3:**
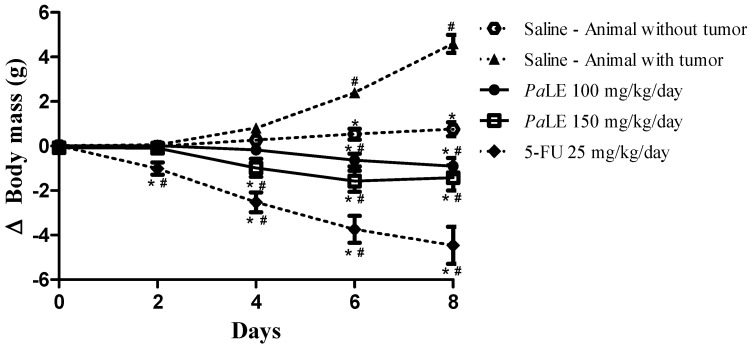
The effect of PaLE (i.p.) on the evaluation of the variation in body mass (g) of mice without and with S180 tumor, for 8 days. Data are presented as the mean ± the standard error of the mean of 10 animals per group. The results were evaluated by two-way ANOVA followed by a Bonferroni post-test. * *p* < 0.05 compared to the group of animals with the tumor, treated with saline; # *p* < 0.05 compared to the group of animals without tumor, treated with saline. Saline was used with negative control and 5-FU as the positive control.

Water consumption, food, and body mass variation were the first parameters to monitor for a period of 8 days. The results obtained demonstrate that the groups of animals treated with *Pa*LE showed a reduction in body mass and food consumption, without altering water consumption (Figure 3 and Table 3). In our study, only the increase in the spleen mass of animals treated with *Pa*LE was observed among the analyzed organs, a potential indication of an immunomodulatory activity (Table 3). Thus, it is possible to state that given the results presented so far that *Pa*LE has cytotoxic activity against the tumor cell line, with insignificant activity against non-tumor cells and antitumor activity in vivo, with a significant reduction in body mass and increased spleen mass, with no changes in liver and kidney mass.

To assess the activity of *Pa*LE on liver and kidney function, ALT, AST, and FA were measured for liver function, and uric acid, urea, and creatinine for renal function (Table 4). When analyzing liver function, no significant changes were found in ALT and alkaline phosphatase measurements. In contrast, when analyzing the AST, an increase in the parameter was found when compared to the group of healthy animals without a tumor and a reduction in the AST when compared to the group of animals treated with tumor saline (Table 4). 

**Figure 4:**
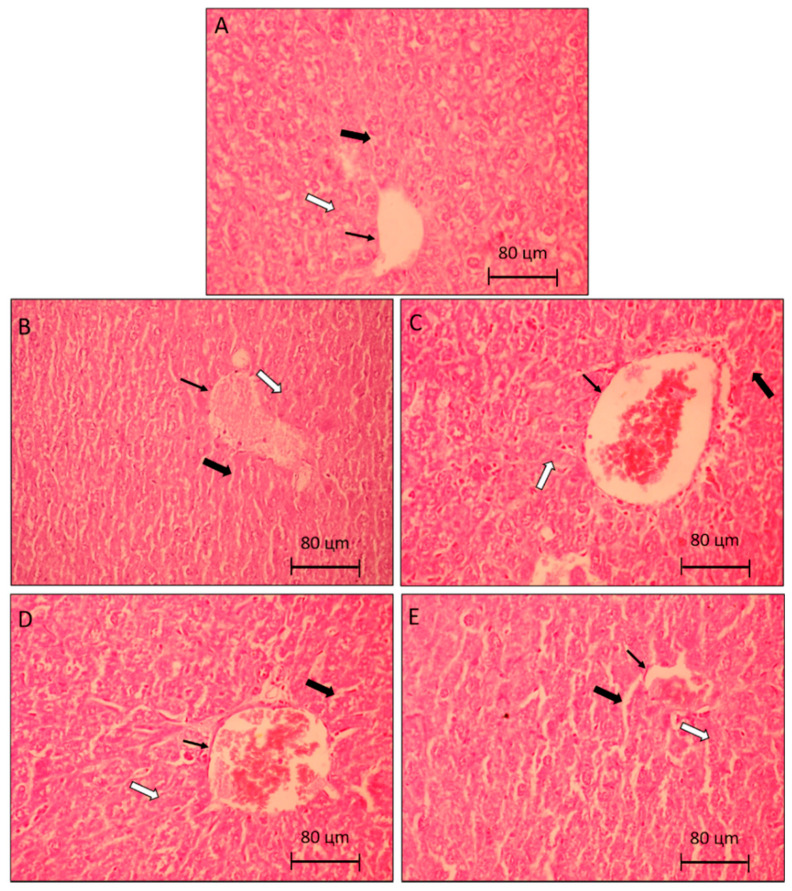
Photomicrographs representative of liver histology (5 µm). Control group (**A**), are animals without tumor S180 treated with the vehicle (saline). The control group (**B**), are animals with tumor S180 treated with the vehicle (saline). Group (**C**,**D**), are animals with S180 tumor treated with PaLE 100 and 150 mg/kg/day, respectively. The positive control group (**E**), are animals with S180 tumor treated with 5-FU 25 mg/kg/day. The thin arrows indicate centrilobular vein, hollow arrows: hepatic trabeculae, and full arrows: sinusoid. 400× magnification.

This result is justified by the fact that AST is also found in skeletal and cardiac muscles, kidneys, pancreas, and erythrocytes. Therefore, the decrease in AST, evidenced in the groups treated with *Pa*LE when compared to the group of animals with tumor saline, may not be related to liver damage, but it is possibly linked to the decrease in tumor size. Similarly, the significant increase in the AST value of the group of animals treated with *Pa*LE, when compared to the group of healthy animals without a tumor, is also not linked to any hepatic alteration, but it is possibly linked to the presence of the tumor in the axillary region. To confirm the hypothesis that *Pa*LE does not cause liver changes in the used dosages, histological cuts were performed. In the analysis of histological photomicrographs of the liver, there was no morphological difference between the groups or any structural alteration. Intact hepatocytes are seen in a row, with nucleus and cytoplasms without morphological alteration (Figure 4). It is confirmed that *Pa*LE does not cause histological changes in the organ due to the adopted protocol. At the tested doses and timeframe, *Pa*LE was shown not to alter the liver mass, as no clinically significant liver parameters, nor histological structures demonstrate otherwise (Table 4 and Figure 4).

**Figure 5:**
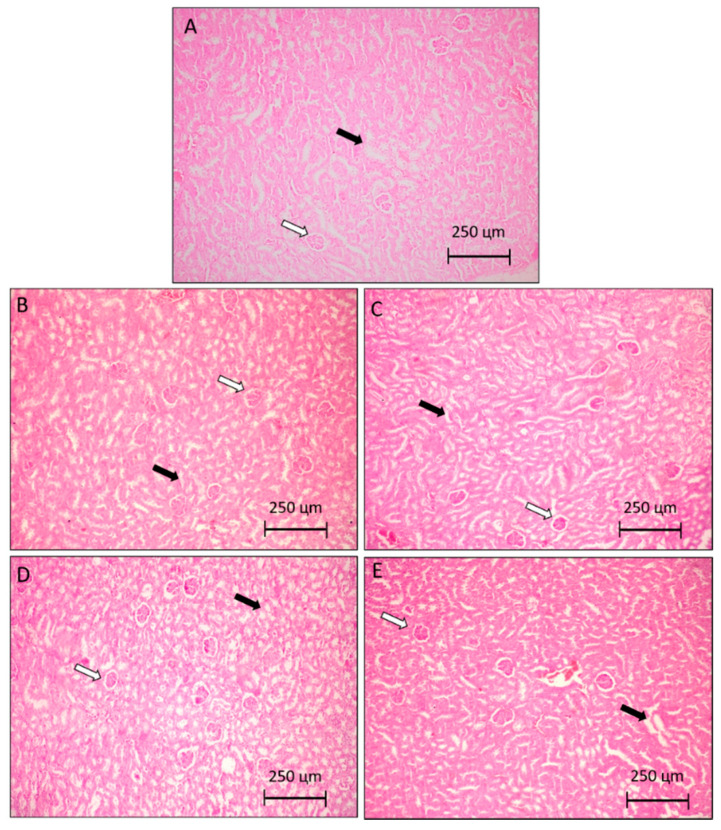
Photomicrographs representative of kidney histology (5 µm). Control group (**A**), are animals without tumor S180 treated with the vehicle (saline). The control group (**B**), are animals with tumor S180 treated with the vehicle (saline). Group (**C**,**D**) are animals with S180 tumor treated with PaLE 100 and 150 mg/kg/day, respectively. The positive control group (**E**), are animals with S180 tumor treated with 5-FU 25 mg/kg/day. The hollow arrows indicate Bowman’s space and arrows filled with preserved proximal twisted tubules. 100× magnification.

When analyzing the renal function of animals undergoing treatment with *Pa*LE, no significant changes were found in the dosages of uric acid, urea and creatinine (Table 4). To confirm the hypothesis that *Pa*LE does not cause renal changes in the dosages used, histological cuts were performed and analyzed, confirming that *Pa*LE does not cause renal histological changes according to the adopted protocol (Table 4 and Figure 5).

The leukocyte hematological parameters evaluated in animals treated with *Pa*LE were the total leukocytes and differential count. Chemotherapeutic agents commonly cause hematological changes and more specifically leukopenia and percentage changes in the lymphocyte and neutrophil ratio in the differential count. This information was confirmed by Britto et al. [5], in which a drastic decrease in the levels of total leukocytes and a change in the percentage ratio of lymphocytes and neutrophils were observed in the group treated with 5-FU.

In our study, the treatment of animals with the 5-FU antineoplastic produced the same response pattern (Table 5), which explains the decrease in the size of the spleen. However, the test groups in our study, treated by i.p. with *Pa*LE, demonstrated a significant increase in the number of total leukocytes (leukocytosis) and a change in the percentage relationship between lymphocytes and neutrophils (Table 5).

**Figure 6:**
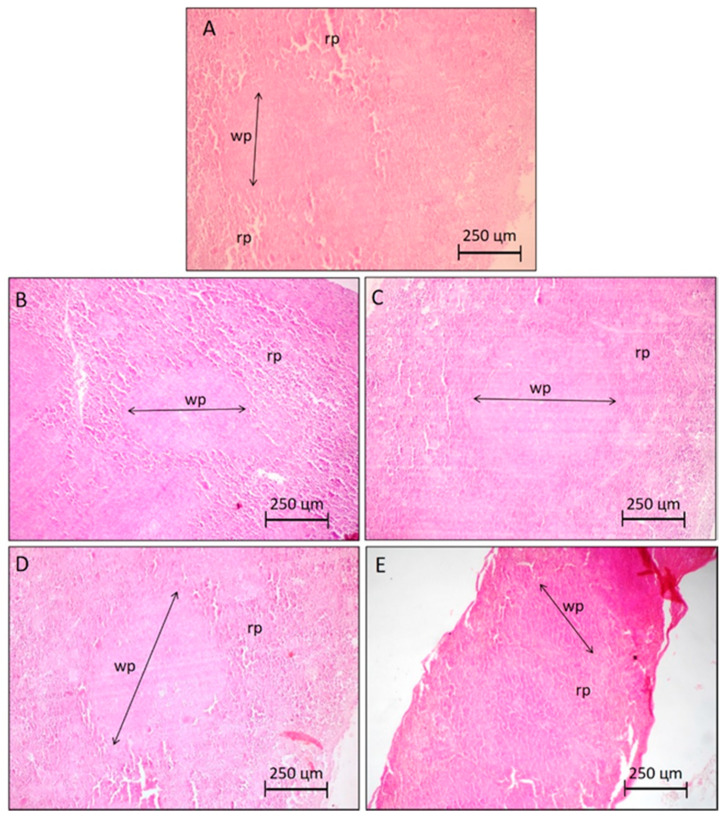
Photomicrographs representative of the spleen histology (5 µm) of groups of animals without and with S180 tumor, highlighting the limits of the white pulp (wp) and red pulp (rp). Control groups (**A**,**B**) are animals without and with tumor S180 treated with the vehicle (saline), respectively. Groups (**C**,**D)** are S180 tumor-bearing animals treated with PaLE 100 and 150 mg/kg/day, respectively. The positive control group (**E**) are S180 tumor-bearing animals treated with 5-fluorouracil 25 mg/kg/day. 100× magnification.

To verify the spleen structure of animals treated with *Pa*LE, histological sections of the spleen were performed and analyzed. In histological photomicrographs of the spleen, of animals treated with saline (A and B), no structural changes were observed. In groups treated with P*a*LE at doses of 100 and 150 mg/kg/day, an increase in white pulp in the spleen was observed. In contrast, Visible atrophy was observed in the spleens of the mice treated with 5-FU (Figure 6). Therefore, according to our results, at the tested doses and timeframe, *Pa*LE changes the spleen mass, the leukocyte hematological parameters and increases the white pulp of the organ. The erythrocyte hematological parameters evaluated in animals treated with *Pa*LE were red blood cells, hemoglobin, and hematocrit; for which no significant changes were observed (Table 5). These results reaffirm the absence of hemolytic activity as previously described (Figure 1).

*Passiflora* genus comprises a diversity of species disseminated in Brazilian regions [23,24]. The plant is endemic in Amazon, with a geographical spread from Peru to eastern Brazil. Its pharmacological properties have been identified and the plant included in the Brazilian Pharmacopoeia in 1929. While several scientific studies describe their pharmacological activities, only a few studies have been published on the antitumor activity of *Passiflora* species. Amaral et al. [20] carried out for the first time an investigation into the cytotoxic potential of the leaves of 14 species of the *Passiflora* genus native to Brazil (*P. alata*, *P. capsularis*, *P. cincinnata*, *P. gibertii*, *P. maliformis*, *P. mallacophylla*, *P. morifolia*, *P. murchronata*, *P. quadrangularis*, *P. racemosa*, *P. setacea*, *P. suberosa*, *P. tenuifila*, and *P. vitifolia*), against human tumor cell lines. Of these, *P. alata* leaf extract showed greater cytotoxic potential as a consequence of the possible synergistic action among its flavonoids.

The presence of saponins in *Pa*LE may be relevant to its antitumor effect and corroborate the findings reported by Amaral et al. [20]. Saponins exhibit various pharmacological effects (e.g., cardiovascular protective activity, anti-inflammatory, antiviral and immunoregulatory effects). A significant anti-cancer activity, with antiproliferative, anti-metastatic, anti-angiogenic and reverses multiple drug resistance (MDR) effects through mechanisms that include apoptosis induction and promotion of cell differentiation, has also been described. Besides, saponins can also reduce the side effects of radiotherapy and chemotherapy, suggesting that this class of compounds is a promising approach for anticancer research [25,26].

For a natural compound to progress as a potential anti-cancer drug candidate, it is required to ascertain the degree of specificity of the drug, through the evaluation of cytotoxic activity in vitro against non-tumor cells [27,28]. In this context, extracts with an CPI > 75% are considered highly cytotoxic and, according to the pre-clinical cytotoxic drug screening program from the United States National Cancer Institute, only extracts with IC_50_ values below 30 μg/mL in trials with tumor cell lines are considered promising for the development of anticancer drugs [28,29].

Silveira et al. [30], demonstrated that the fraction of *Pa*LE enriched with saponin shows little or no hemolytic activity in the tested concentrations, which corroborates the result reported by us. Dai et al. [31] described that the presence of flavonoids in the crude extract may explain the insignificant hemolytic activity found in *Pa*LE, when compared to some isolated saponins. It is known that some flavonoids and their glycosides are effective antioxidants and that they protect erythrocytes from oxidative hemolysis induced by free radicals [31].

Currently, most drugs used in chemotherapy are administered parenterally, with limited oral drug dosage forms [32]. The administration of antineoplastic agents by the oral route is indeed associated with a higher risk of toxicological events, including irritability of the walls of the stomach and intestine, and consequently a slower and incomplete absorption, leading to lower bioavailability [5]. These may justify the results obtained by the oral administration of *Pa*LE, which consequently compromised its activity.

Although *P. alata* has not been previously investigated for its antitumor activity, the cytotoxic, antiproliferative, chemopreventive, and/or antitumor activity of several *Passiflora* species against cancer cells have been already described in the scientific literature. The cytotoxic or antiproliferative activity of species of the genus *Passiflora* was evidenced for the first time by Perry et al. [19], who described the cytotoxic activity of the extract of *P. tetandra* leaves against cells of the leukemic lineage of murine (P388), possibly mediated by a component of the extract called 4-hydroxy-2-cyclopentane. The ethanolic extract of *P. ligularis* Juss. and the methanolic extract of the fruit of *P. edullis* were also described for their activity against the human hepatocellular carcinoma (Hep3B) and the leukemic cell line (CCRF-CEM), respectively [13,14].

The compound called chimaphilin (2,7-dimethyl-1,4-naphthoquinone), isolated from *P. incarnata*, exhibited cytotoxic activity against the human breast cancer strain (MCF-7) [16]. The chemopreventive activity was found in vitro for the ethanol extract of *P. incarnata* and in vivo (colorectal cancer model) in the consumption of the fruit of *P. molissima* [15,18]. The ethanolic extract of *P. incarnata* was reported to have a significant antitumor activity against Ehrlich ascitic carcinoma model [17]. *P. alata* is the first species of the genus to be described with cytotoxic activity in vitro and antitumor in vivo.

It is known that most of the antineoplastic drugs currently used are cytotoxic to tumor cells, but also have nonspecific action, as they reach normal cells, which leads to many undesirable side effects [33]. Therefore, the search for compounds that can reduce the harmful side effects of chemotherapy in normal tissues is in demand. Bearing this in mind, we have investigated possible toxicological aspects of *Pa*LE in vivo at doses of 100 and 150 mg/kg/day.

Loss of body mass and reduced food intake were also reported in a study published by Braga et al. [34], upon the oral administration of 250 mg/kg/day of aqueous extract of *P. alata* leaves over a period of 14 days. These findings may be a sign of toxicity or may be related to possible anorectic properties of the species.

In the evaluation of the toxicological parameters of potential drug candidates with antitumoral activity, spleen, liver and kidneys are also evaluated for changes in their mass, as they are organs susceptible to the effects of antineoplastic agents [35].

Potential activity against neoplasms with an increase in spleen mass has also been reported for the hydroalcoholic extract of *Remirea maritime* and the ethanolic extract of the bark of *Himatanthus drasticus* [36,37]. This property presented by *Pa*LE can be considered beneficial because it counteracts the adverse effect that occurs with many available antineoplastic agents (spleen retraction), reflecting on the immunosuppressive action of these drugs, which is a limiting factor in cancer therapy. 5-FU is within this class of drugs, leading to a reduction in spleen mass as shown in Table 3 [38,39].

The S180 tumor, in its solid form and as it grows in the axillary region, damages one or more tissues, such as skeletal muscle; a factor that causes changes in biochemical parameters such as AST [38]. For this reason, care should be taken with isolated elevation of the AST, as it does not correspond, in most cases, to liver damage.

When assessing liver toxicological parameters, these outcomes shown by *Pa*LE are of great relevance since the liver is the largest and most complex of internal organs and collateral damage caused in cancer therapy is not uncommon. Hepatotoxicity from chemotherapy occurs frequently, and pre-existing liver disease increases this risk. The pattern of the presentation can vary from inflammatory hepatitis, cholestasis, steatosis, and, finally, occlusive hepatic venous disease. The severity varies from an asymptomatic elevation of the liver function test, acute liver failure, or progressive fibrosis that culminates in end-stage liver disease. Several antineoplastic drugs, such as etoposide and vincristine, produce liver toxicity [40].

A study with longer administration may show some hepatic alteration, as demonstrated by Braga et al. [34] with aqueous extract of *P. alata* leaves, administered for 14 days, in doses of 25 and 250 mg/kg/day, which induced a reduction in liver mass, a reduction in ALT and a hydrological histological degeneration. Given the fact that the liver results reported by Braga et al. [34] were obtained with 3 to 5 animals per group, longer and more detailed studies may be of scientific interest. In contrast, another study by Rudnicki et al. [41], in which extract of *P. alata* leaves was administered to rats, demonstrated the hepatoprotective potential of the extract in the dosages of 1 and 5 mg/kg/day after 30 consecutive days of administration. Therefore, a more specific study is needed, with varying periods and doses of administration for a consistent understanding of possible liver changes.

Antineoplastic drugs commonly cause immunosuppression as an adverse effect and consequently substantially increase the risk of infection in cancer patients undergoing chemotherapy [38]. The results obtained with our *Pa*LE may mean an advantage over some antineoplastic drugs because it does not promote a potential immunosuppressive activity and consequently minimizes the possibility of infectious events.

The work carried out by Silveira et al. [30] strengths the perspective of the absence of immunosuppressive activity and a potential immunomodulatory activity, since their results demonstrated that the saponin found in *P. alata* may have potential immunostimulatory action. Also, a literature review published by Patel et al. [42] demonstrates that diosmetin, a compound present in *Pa*LE, can influence the increase in the lymphocyte rate.

A similar result was found by Dória et al. [36] with the hydroethanolic extract of *Remirea maritime*, who demonstrated that the spleen of the treated animal was enlarged, the number of leukocytes elevated and the white pulp of the organ increased, being indicative of possible immunostimulatory activity. It is thus suggested that *Pa*LE can exhibit immunomodulatory activity, however more specific tests with *Pa*LE should be performed, to generate data that can better outline these effects.

## 3. Materials and Methods

### 3.1. Source of Leaf Extract from Passiflora alata Curtis

The leaves of the species *P. alata* Curtis were collected on 22 July 2016, at the Brazilian Agricultural Research Corporation (EMBRAPA), in Cruz das Almas (Bahia, Brazil). The sample was subjected to drying in a circulating air oven for seven days, at 30 °C. After that period, it was packed in transparent plastic bags and duly identified. The dry vegetable material was sprayed in a blender and the granulometry was standardized with a 16 mesh sieve (1.00 mm mesh), obtaining 96.88 g. *Passiflora* leaves were pulverized and submitted to extraction using an accelerated solvent extraction device (ASE 100Dionex corporation, Sunnyvale, CA, USA), equipped with a 34 mL stainless steel extraction cell, with hermetic closure and 22 × 50 mm paper cartridge, a washing bottle (rinse) and collecting bottles (transparent glass) with a capacity of 250 mL. The extract was prepared by weighing exactly 6.0 g of the *P. alata* powder from the plant material, was transferred to the 34 mL extraction cell and subjected to extraction with the ethanol: water (64:36 *v*/*v*) system, oven temperature of 80 °C, 5 extraction cycles, extraction time of 10 min, 100% flushing, under the pressure of 1500 psi (N_2_), for 60 s. Subsequently, each extract was concentrated under reduced pressure in a rotary evaporator at 55 °C, until the complete removal of the solvent, resulting in the respective crude extracts, with a yield of 48.2%. The extracts were transferred to capped glass bottles, identified, weighed, and kept in a freezer.

### 3.2. Saponin Identification

To prepare the extracts, fractions, and compounds for analysis by UPLC (Waters, Milford, MA, USA), 2.0 mg of the sample was weighed, which was subsequently dissolved in 1 mL of methanol in Eppendorf flasks, followed by ultrasonic sonication for 20 min. Then, the sample was centrifuged at 10,000 rpm, for 10 min, with subsequent filtration in 22 μm filtration membranes. For all samples, the supernatant was used in the analysis by UPLC-DAD. In all stages, UPLC grade solvents and distilled water filtered in a Milli-Q system (home supplied), were used.

To obtain the UPLC-DAD chromatographic profiles, a Acquity Ultra Performance LC chromatograph, PDA eʎ, and TQ Detector (Waters, Milford, MA, USA) An RP-18 Acquity UPLC BEH column (particles 2.1 × 50 mm, 1.7 μm), flow 0.3 mL/min was used and the column oven was maintained at 40 °C. The sample injection volume was 4 μL, with gradient elution with H_2_O (0.1% HCOOH)/CH_3_CN (0.1% HCOOH). The chromatographic parameter used comprised a period of linear elution (5–95% ACN from 0 to 10 min), maintaining than a short period of isocratic elution (95% CH_3_CN from 10 to 11 min), returning to the condition of initial elution 11–13 min (5% CH_3_CN).

The mass spectra were obtained with electron spray ionization (ESI) and recorded in the full scan (full scan) and sequential or tandem (MS/MS), in positive and/or negative modes in the Waters ACQUITY^®^ TQD equipment, equipped with a quadrupole analyzer. The general operating conditions of the equipment during the analyzes were: capillary voltage: 3.5 kV; capillary temperature: 320 °C; desolvation temperature: 320 °C; collision gas: argon, cone voltage: 5 kV; ionization voltage: −4 kV; orifice voltage: −60 kV. The samples were injected by an automatic injection pump with a continuous flow of 0.1 μL/min. The ESI/EM/EM spectra were recorded with energy of 30 eV in the range of *m/z* 100–2000 u.m.a.

### 3.3. Tumor Cells

To evaluate the cytotoxic activity of *P. alata*, four tumor cell lines were used: Prostate carcinoma (PC-3), Hepoc2 cellular carcinoma (HepG2), melanoma (B16-F10) and sarcoma 180 (S180). All tumor cell lines were provided by the National Cancer Institute (Bethesda, MD, USA) or acquired through the Rio de Janeiro cell bank. The cells were grown in cell culture bottles (75 cm^3^, 250 mL volume), the media used were RPMI 1640 and supplemented with 10% fetal bovine serum and 1% antibiotics (penicillin/streptomycin). The cells were kept in incubators with an atmosphere of 5% CO_2_ at 37 °C. Cell growth was monitored daily using an inversion microscope. The medium was changed whenever the cell growth reached the confluence necessary for nutrient renewal. Cell cultures were negative for mycoplasma, as assessed by Hoechst placement (Mycoplasma Stain Kit, Cat. MYC1, Sigma-Aldrich^®^, St. Louis, MO, USA).

### 3.4. Cytotoxicity in Tumor Cell Lines

To determine the cytotoxic potential of the extract against tumor cell lines, the 3-(4,5-dimethyl-2-thiazol)-2,5-diphenyl-2-H-tetrazolium bromide (MTT) salt method was used [43].

For all experiments, in a 96-well plate, tumor cell lines were plated (100 µL/well) at concentrations of 0.7 × 10^5^ cells/mL (HepG2 and B16-F10) and 0.1 × 10^6^ cells/mL (PC-3). After 24 h, *PaLE* was dissolved in 0.3% dimethyl sulfoxide (DMSO) and added to each well in a single concentration of 50µg dry crude extract/mL. The experiment was carried out in three independent moments in triplicate, using 0.25 µg/mL doxorubicin and 0.3% DMSO as positive and negative control, respectively. The plates were incubated for 72 h in an oven with 5% CO_2_, at 37 °C.

At the end of the incubation, the plates were centrifuged (15/15 min) at 4 °C and the supernatants removed. Then, 150 µL of the MTT solution (0.5 mg/mL) was added, and the plates were incubated for 3 h. After that period, the plates were again centrifuged (30 g/10 min) at 4 °C, the supernatants discarded and the precipitates resuspended in 150 µL of pure sterile DMSO. For the quantification of formazan by viable cells the absorbance was read using a multiplate reader (DTX 880 Multimode Detector, Beckman Coulter Inc., Packard, ON, Canada) at 595 nm. All values were converted to percentage of cell growth inhibition (% CPI) using the following equation:

$$\mathrm{CPI}\;\left(\%\right)=100-\left[\left(\frac{T}{NC}\right)\times 100\right]$$

where *T* stands for *Pa*LE absorbance and *NC* stands for absorbance of the negative control. The results were analyzed using a CPI (%) scale as follows: samples with low activity have CPI < 50%, moderate activity has CPI between 50% and 75% and high activity has CPI > 75% for each cell line tested [29]. To determine the inhibitory concentration capable of causing 50% of its maximum effect (IC_50_), the same protocol previously described for CPI (%), with the same cell lines, was performed, varying only the *Pa*LE concentration from 0.39 µg/mL to 50 µg/mL.

### 3.5. Hemolytic Activity

To assess the potential of *Pa*LE to cause lesions (formation of pores or rupture) in the plasma membrane of erythrocytes, five Swiss mice (*Mus musculus*) were subjected to transient anesthesia with isoflurane (1.5%; via inhalation), using a vaporizer calibrated coupled to an oxygen gas cylinder in an exhausted environment (chapel), for blood collection through the introduction of a glass capillary in the orbicular region of the eye.

To obtain the erythrocyte solution, 2 mL of whole blood was diluted in 10 mL of saline solution (0.85% NaCl + 10 mM CaCl_2_) and then centrifuged at 1500 rpm for 5 min. The supernatant was removed, discarded and the pellet was resuspended in saline, and the process was repeated 2 more times. The erythrocytes were resuspended in saline to obtain a 2% erythrocyte solution to be used in the hemolysis assay.

The tests were performed in 96-well multi-plate, in triplicate. Each well in the first row received 100 µL of saline (negative control). In the second row, the wells received 100 µL of 1% Triton X-100 (positive control). In the third, fourth, fifth, and sixth row, *Pa*LE diluted in saline in concentrations of 125, 250, 500, and 1000 µg dry crude extract/mL respectively. Then 100 µL of the erythrocyte suspension was plated in all wells.

After incubation for 60 min, under constant agitation and at room temperature (25 ± 2 °C), the plates were centrifuged at 1500 rpm for 10 min, the supernatant carefully removed and transferred to another plate to read the absorbance of the amount of released hemoglobin. In the supernatant in a spectrophotometer at 540 nm (Kang et al., 2009; Pita et al., 2012). All values were converted to the percentage of hemolysis using the following equation:

$$\mathrm{Hemolysis}\;\left(\%\right)=\;\left(\frac{T}{NC}\right)\times 100$$

where *T* stands for *Pa*LE absorbance and *NC* stands for absorbance of the negative control.

### 3.6. Antitumoral Activity In Vivo

For the study of antitumor activity in vivo, the experimental transplantable tumor called Sarcoma 180 tumor (S180) or Crocker tumor was used. The tumor cells were obtained from the Experimental Oncology Laboratory of the Federal University of Ceará and maintained in their ascitic form in the abdominal cavity of Swiss mice (*Mus musculus*), every 15 days, at the Pharmacology Laboratory of the Inflammatory Process of the Federal University of Sergipe.

The study was conducted in accordance with the Declaration of Helsinki, and the protocol was approved by the Ethics Committee on Animal Research of the Federal University of Sergipe (Project identification code CEPA 27/2015, date 24 November 2015). The animals were housed in polypropylene boxes with appropriate metal grids in small groups of 10 mice of the same sex. The temperature of the place was 21 ± 2 °C and the relative humidity was 30–70%. The lighting was artificial, alternating 12 h of light and 12 h of darkness. The animals had free access to food and water.

The maintenance mouse or donor was anesthetized with isoflurane (1.5%; via inhalation), using a calibrated vaporizer coupled to an oxygen gas cylinder in an exhausted environment (chapel) and euthanized by cervical dislocation. The aseptic procedure of the abdominal region was performed with iodized alcohol and 0.5 mL of ascitic fluid was collected from the animal’s abdominal cavity. After collection, a cell suspension was prepared with 5.0 mL of lactated ringer, 0.2 mL of gentamicin (5 mg/mL), and the volume of ascitic fluid collected, for later cell counting. Healthy recipient animals were inoculated with 2 × 10^6^ cells/0.5 mL intraperitoneally (i.p.).

### 3.7. In Vivo Antitumoral Activity in Sarcoma Tumor 180-Bearing Mice

To assess antitumor activity in vivo, the donor or maintenance mouse, with 10 days of growth of the ascitic S180 tumor, was previously anesthetized and euthanized by cervical dislocation. After asepsis with iodized alcohol in the abdominal region, ascitic fluid was removed from the abdominal cavity of the mouse, with the aid of a 5 mL syringe. Then, a cell suspension was made with 5 mL of lactated ringer, 0.2 mL of gentamicin (5 mg/mL) and 0.5 mL of the ascitic tumor. From this suspension, an aliquot of 50 µL was removed and 50 µL of trypan blue was added for counting in Neubauer chamber, viable tumor cells. The non-viable cells were stained blue due to the inability to pump the trypan out of the cell, and the viable ones without staining.

From the viable cell count, 2 × 10^6^ cells/0.5 mL/mouse were inoculated into the recipient animals, subcutaneously, in the animal’s left axillary region [44,45,46]. 24 h after inoculation, treatments were started to assess the effect of *Pa*LE on tumor growth; one application a day for 7 consecutive days. A total of 100 animals were used, distributed in 10 groups of 10 animals in polypropylene boxes, 50 of which (5 groups) received intraperitoneal (i.p.) and 50 animals orally. Animals that received i.p. were distributed as follows: (1) negative control (0.9% saline), (2) *Pa*LE 50 mg/kg/day, (3) *Pa*LE 100 mg/kg/day, (4) *Pa*LE 150 mg/kg/day and (5) positive control (5-Fluorouracil 25 mg/kg/day, 5-FU). The animals that received oral treatment were distributed as follows: (1) negative control (0.9% saline), (2) *Pa*LE 100 mg/kg/day, (3) *Pa*LE 200 mg/kg/day, (4) *Pa*LE 300 mg/kg/day and (5) positive control (cyclophosphamide 15 mg/kg/day, Cyclo).

After 24 h of the last day of treatment, the animals were submitted to transient anesthesia with isoflurane (1.5%; via inhalation), using a calibrated vaporizer coupled to an oxygen gas cylinder in an exhausted environment (chapel), for collection of blood through the orbicular region of the eye (6 animals per group), for the analysis of biochemical and hematological parameters. Then, all the animals were euthanized by cervical dislocation and the tumor, liver, kidneys, and spleen were removed, weighed and preserved, in 10% formaldehyde. The percentage of tumor growth inhibition (TGI (%)) was calculated using the following equation:

$$\mathrm{TGI}\;\left(\%\right)=\;\left[\frac{A-B}{A}\right]\times 100$$

where *A* stands for the mean tumor mass in the negative control group, and *B* for average tumor mass in the treated animals.

### 3.8. Evaluation of the Toxicological Effects

The toxicological effects of *Pa*LE were carried out in the groups of test animals that showed antitumor activity, their respective controls and added a group of 10 healthy mice (without inoculation of the S180 tumor).

#### 3.8.1. Determination of Body Mass

All groups of animals under experimentation, before treatment administration, were subjected to body mass verification on an analytical balance on days 0, 2, 4, 6 and 8.

#### 3.8.2. Determination of Water and Feed Ingestion

On the first day of inoculation of the S180 tumor, and 24 h after the last day of treatment, the amounts of food and water ingested during the experiments were determined.

#### 3.8.3. Determination of the Mass of Organs

After euthanasia of healthy animals inoculated with S180 tumor, the spleen, liver and kidneys were removed, examined macroscopically for changes in color, presence of hemorrhage, necrosis and other changes. The mass of the organs was also determined by their weighing on an analytical balance and standardized for 100 g of body mass, as described by the following equation:

$$\mathrm{Organ}\;\mathrm{mass}\;\left(g/100\;g\right)=\;\frac{\mathrm{MO}\;\left(g\right)}{\mathrm{ABM}\;\left(g\right)}\;\times 100\;\left(g\right)$$

where MO stands for the mass of organs and ABM for the animal body mass.

### 3.9. Evaluation of Biochemical Parameters

Among the existing biochemical parameters, those that may reflect possible problems in the liver and kidney functions of animals submitted to treatments were evaluated. The tests performed for liver evaluation were alanine aminotransferase (ALT) formerly called glutamic-pyruvic transaminase (TGP), aspartate aminotransferase (AST) formerly called glutamic oxalacetic transaminase (TGO) and alkaline phosphatase (FA). For renal evaluation, the biochemical parameters used were uric acid, creatinine, and urea. The analyzes were performed using blood collected via the orbital plexus with the aid of a heparinized cannula, 24 h after the last day of treatment of animals with an S180 tumor. The samples were collected in a dry tube, submitted to centrifugation for 10 min, with 3500 rotation per minute (rpm), at 25 °C to obtain the serum used for biochemical measurements in an automated analyzer (Architect C 8000, Abbott, Chicago, IL, USA) with kits from Clinical Chemistry.

### 3.10. Evaluation of Hematological Parameters

Amongst the existing hematological parameters, leukocyte (total and differential leukocytes) and erythrocyte (red blood cells, hemoglobin, and hematocrit) parameters were evaluated. The total leukocyte count was performed with 20 µL of blood, collected through a heparinized cannula via the orbital plexus of animals with an S180 tumor, 24 h after the last day of treatment and diluted in 380 µL of Turk. Then, a volume of 10 µL of the suspension was placed in a Newbauer chamber and total leukocytes counted. The differential leukocyte count was performed employing a drop of whole blood added to a clean slide and identified for the preparation of blood smears. Smear staining was performed using panoptic dyes (Pinhais, Brazil, Instant-Prov^®^) and the identification of 100 leukocyte cells (basophils, eosinophils, lymphocytes, monocytes, and neutrophils) performed using an optical microscope. To evaluate the erythrocyte hematological parameters (red blood cells, hemoglobin, and hematocrit), whole blood was collected through a heparinized cannula via the orbital plexus of animals with an S180 tumor, 24 h after the last day of treatment. The blood was placed in a tube containing the EDTA anticoagulant for the evaluation of erythrocyte parameters in an automated analyzer (BC5380, Mindray, Shenzhen, China).

### 3.11. Histopathological Evaluation of Organs

Organs (spleen, liver, and kidneys) were sectioned after their weighing, fixed in formalin (10% formaldehyde solution) and, after 72 h they were resected for histopathological processing as follows: dehydration with an increasing ethanolic series (70, 80, 90, 95 and 100%; 50 min each) and diaphanized in three 50-min baths (xylol I, xylol II, xylol III). In the inclusion step, aluminum capsules filled with the organ cut along with liquefied paraffin were used for heating. Material was allowed to solidify upon cooling down, being retained inside the solidified paraffin. In a semiautomatic rotary microtome (Buffalo, NY, USA, American Optical^®^), the fragments embedded in paraffin were then sectioned in a thickness of 5.0 μm, collected with glass slides in a water bath and stored in the oven at 37 °C. After 24 h, the deparaffinization, hydration, and hematoxylin-eosin (HE) staining step started.

## 4. Conclusions

In conclusion, *Pa*LE was shown in vitro cytotoxic activity, with promising uses in the development of antitumor drugs against PC-3 and S180, without presenting in vitro toxicity against red blood cells, with antitumor activity in a preclinical model of S180 tumor by intraperitoneal route. The toxicological parameters were found normal, except for toxicity related to possible anorexigenic properties. These activities are possibly mediated by the synergistic action of the constituents found in the extract.

## Figures and Tables

**Table 1:** Partial identification of saponins in *Pa*LE by UPLC-DAD.

Compounds	RT (min)	[M + H]^+^ (*m/z*)	[M − H]^−^ (*m/z*)	[M + CHCOOH]^−^ (*m/z*)	Sugar Residues	Characteristic *m/z* of Ions in Negative Ion Mode
C_42_H_68_O_13_	6.15	781	779.6	825	Glc; Glc	779.2; 617.5; 455.1
C_42_H_68_O_13_	6.23	781	779.8	825	Glc; Glc	779.4; 617.4; 455.2
C_42_H_70_O_13_	6.86	783	781.5	827	Glc, Glu	781.5; 619.7; 443.3; 178.8; 161.1
C_42_H_70_O_13_	6.07	783	781.8	827	Glc; Rham	781.4; 619.6; 160.8; 146.8
C_42_H_70_O_13_	6.48	783	781.5	827	Glc; Pent	781.5; 619.0; 161.3; 130.9
C_42_H_70_O_14_	4.81	799	797.4	843	Glc	797.5; 635.2; 179.0
C_42_H_70_O_14_	5.22	799	797.6	843	Glc; Glu	797.6; 635.2; 460.1; 159.3
C_42_H_70_O_14_	5.47	799	797.4	843	Glc	797.4; 635.3; 455.5; 159.6
C_42_H_70_O_14_	6.02	799	797.4	843	Glc	797.3; 635.3; 422.4; 161.2
C_42_H_70_O_15_	5.37	815	813.7	859	Glc; Glc	813.4; 651.2; 489.6; 161.0
C_48_H_80_O_18_	4.96	945	943.5	989	Glc; Glc	943.7; 780.4; 653.2; 621.8
C_48_ H_80_ O_18_	5.01	945	943.6	989	Glc; Glu	943.5; 781.3; 588.8
C_48_H_80_O_19_	4.44	961	959.8	1005	Glc; Glc; Glu	959.2; 797.5; 459.0; 161.2
C_54_H_90_O_23_	4.66	1107	1105.3	1151	Rham; Glu; Glc; Pent	1105.2; 959.8; 782.2; 489.0; 179.0
C_54_H_90_O_23_	4.86	1107	1105.1	1151	Glc; Pent	1105.3; 943.3; 471.4; 131.0
C_54_H_90_O_24_	4.46	1123	1121.4	1167	Glc	11,121.4; 959.1
C_54_H_90_O_24_	4.26	1123	1121.6	1167	Glc	11,121.6; 959.4; 691.4

Glc = glucose/galactose; Glu = glucoronic acid; Rham = rhamnose; Pent = pentose.

**Table 2:** Percentage of inhibition of cell proliferation (% CPI) and the average inhibitory concentration capable of promoting 50% of the maximum effect (IC_50_) of *Pa*LE against four tumor cell lines.

Cell Lines	*Pa*LE		Doxorrubicin	
	% CPI ± SD	IC_50_	% CPI ± SD	IC_50_
PC-3	83.26 ± 2.29	20.24 (16.58–24.70)	98.21 ± 0.54	0.55 (0.39–0.83)
K-562	76.24 ± 3.71	37.92 (29.56–48.63)	99.15 ± 0.15	0.70 (0.53–0.94)
HepG2	75.56 ± 4.08	>50.00	85.70 ± 0.88	0.35 (0.28–0.44)
S180	79.11 ± 2.97	19.13 (15.43–24.96)	81.73 ± 1.18	4.5 (3.33–5.13)

The % CPI values were presented as the mean ± standard deviation and the IC_50_ values in µg/mL with a 95% confidence interval obtained by non-linear regression. The experiments were carried out independently in triplicate after 72 h of incubation. Doxorubicin was used as a positive control.

**Table 3:** Effect of *Pa*LE (i.p.) on the consumption of water, feed and organ mass of mice without and with S180 tumor.

Parameters	Healthy	Animal with Tumor			
	Saline	Saline	*Pa*LE 100 mg/kg/day	*Pa*LE 150 mg/kg/day	5-FU 25 mg/kg/day
Water consumption (mL)	46.2 ± 1.33	41.3 ± 3.70	41.60 ± 0.93	40.90 ± 1.10	15.60 ± 0.40 * ^#^
Feed Consumption (g)	37.05 ± 0.38	35.60 ± 0.63	28.35 ± 0.42 * ^#^	27.95 ± 0.95 * ^#^	17.95 ± 0.78 * ^#^
Spleen (g/100 g m.c.)	0.39 ± 0.01 *	0.52 ± 0.03	0.64 ± 0.02 * ^#^	0.62 ± 0.04 * ^#^	0.29 ± 0.03 * ^#^
Liver (g/100 g m.c.)	5.33 ± 0.12	5.41 ± 0.08	5.64 ± 0.20	5.88 ± 0.15	5.33 ± 0.09
Kidneys (g/100 g m.c.)	1.35 ± 0.03	1.44 ± 0.06	1.33 ± 0.04	1.38 ± 0.04	1.40 ± 0.05

The data are presented as the mean ± the standard error of the mean of 10 animals. The results were evaluated by one-way ANOVA followed by *Student Newman Keuls* post-test. * *p* < 0.05 compared to the group of animals with tumor, treated with saline; # *p* < 0.05 compared to the group of healthy animals, treated with saline. Saline was used with negative control and 5-FU as positive control.

**Table 4:** Effect of *Pa*LE (i.p.) on the hepatic and renal biochemical parameters of the blood of mice with and without S180 tumor.

Parameters	Healthy	Animal with Tumor			
	Saline	Saline	*Pa*LE 100 mg/kg/day	*Pa*LE 150 mg/kg/day	5-FU 25 mg/kg/day
ALT (U/L)	53.60 ± 1.97	51.60 ± 5.08	48.40 ± 4.21	49.67 ± 6.01	41.60 ± 6.00
AST (U/L)	85.75 ± 1.05 *	196.2 ± 9.43 ^#^	147.5 ± 10.26 * ^#^	156.5 ± 11.15 * ^#^	93.33 ± 8.7 *
FA (mg/dL)	70.35 ± 1.97	64.32 ± 4.36	72.55 ± 1.37	69.74 ± 2.79	76.55 ± 3.53
Uric acid (mg/dL)	2.81 ± 0.49	2.67 ± 0.57	2.51 ± 0.35	2.59 ± 0.43	2.78 ± 0.55
Creatinine (mg/dL)	0.32 ± 0.06	0.29 ± 0.01	0.31 ± 0.01	0.28 ± 0.01	0.37 ± 0.02
Urea (mg/dL)	46.60 ± 1.54	42.00 ± 4.35	36.60 ± 3.97	39.00 ± 0.58	49.00 ± 5.86

Data are presented as the mean ± the standard error of the mean of 6 animals. The results were evaluated by one-way ANOVA followed by *Student Newman Keuls* post-test. * *p* < 0.05 compared to the group of animals with tumor, treated with saline; # *p* < 0.05 compared to the group of animals without tumor, treated with saline. Saline was used as a negative control and 5-FU as a positive control.

**Table 5:** Effect of *Pa*LE (i.p.) on hematological parameters of peripheral blood in mice with and without S180 tumor.

Parameters	Healthy	Animal with Tumor			
	Saline	Saline	*Pa*LE 100 mg/kg/day	*Pa*LE 150 mg/kg/day	5-FU 25 mg/kg/day
Total leukocytes (10^3^ cels/µL)	7.29 ± 0.76 *	9.49 ± 0.37 ^#^	12.48 ± 0.55 * ^#^	13.84 ± 0.95 * ^#^	2.40 ± 0.37 * ^#^
Lymphocytes %	69.63 ± 1.19 *	48.13 ± 2.36 ^#^	55.23 ± 4.76 * ^#^	54.40 ± 2.62 * ^#^	82.40 ± 1.29 * ^#^
Monocytes %	1.75 ± 0.53	1.63 ± 0.32	1.17 ± 0.31	1.60 ± 0.24	1.20 ± 0.20
Neutrophils %	28.62 ± 1.13 *	50.25 ± 2.46 ^#^	43.60 ± 3.84 * ^#^	44.00 ± 1.70 * ^#^	16.40 ± 1.21 * ^#^
Red cells (10^6^/mL)	7.83 ± 0.60	7.67 ± 0.61	8.00 ± 0.37	8.17 ± 0.79	7.50 ± 0.56
Hemoglobin (g/dL)	14.17 ± 0.54	13.17 ± 0.79	13.33 ± 0.92	13.50 ± 1.02	12.83 ± 1.14
Hematocrit (%)	43.17 ± 1.72	42.83 ± 1.70	43.67 ± 1.23	44.33 ± 2.17	42.67 ± 1.56

Data are presented as the mean ± the standard error of the mean of 6 animals. The results were evaluated by one-way ANOVA followed by *Student Newman Keuls* post-test. * *p* < 0.05 compared to the group of animals with tumor, treated with saline; # *p* < 0.05 compared to the group of animals without tumor, treated with saline. Saline was used as a negative control and 5-FU as a positive control.
